# Effects of Chinese herbal feed additives on the sperm quality and reproductive capacity in breeding boars

**DOI:** 10.3389/fvets.2023.1231833

**Published:** 2023-07-26

**Authors:** Weilong Tu, Weiyi Zhang, Hongyang Wang, Yingying Zhang, Ji Huang, Bushe Li, Xin Li, Yongsong Tan, Xiao Wu

**Affiliations:** ^1^Institute of Animal Science and Veterinary Medicine, Shanghai Academy of Agricultural Sciences, Key Laboratory of Livestock and Poultry Resources (Pig) Evaluation and Utilization, Ministry of Agriculture and Rural Affairs, Shanghai, China; ^2^Institute of Shanghai Engineering Research Center of Breeding Pig, Shanghai, China; ^3^Shanghai Center of Agri-Products Quality and Safety, Shanghai, China; ^4^Biotechnology Research Institute, Shanghai Academy of Agricultural Sciences, Shanghai, China

**Keywords:** traditional Chinese medicine, additive, breeding boars, sperm quality, serum hormone, reproductive capacity

## Abstract

Currently, Chinese herbal feed additives (CHFA) are commonly utilized in domestic pig farms. However, their impact on the sperm quality and reproductive capacity of imported breeding boars has yet to be thoroughly explored. In this study, the effect of CHFA on the sperm quality and reproductive capacity of the imported Duroc boars was investigated. Sixteen boars were randomly divided into control group and experimental (CHFA treated) group and fed normal or CHFA-levels containing diets, respectively. The sperm quality and reproductive hormone levels were periodically tested, and the reproductive capacity with breeding sows were evaluated. The results showed that the CHFA treated group boars significantly improved sperm volume, sperm concentration, and motility and reduced the sperm abnormalities. Furthermore, the serum levels of reproductive hormone such as follicle-stimulating hormone (FSH), luteinizing hormone (LH), and testosterone (T) in the CHFA treated group were significantly higher than those in the control group. Although there was no significant difference in the initial birth weight of piglets between the two groups, the CHFA treated group had a significantly higher average number of piglets born, the average number of piglets born alive, the number of piglets weaned at 28 days, and the weaning weight compared to the control group. These findings suggest that CHFA can significantly improve the sperm quality of breeding boars and enhance their reproductive hormone levels as well as the reproductive capacity, providing direct evidence for the further application of CHFA in the management of breeding boars in China.

## 1. Introduction

Chinese herbal medicine (CHM) is a traditional medicinal practice that utilizes various plant parts and extracts for therapeutic purposes, they are known for their medicinal properties (1–4), natural ingredients (5, 6), and diverse functions (1, 7), making the CHM used as a promising adjunctive therapy and feed additive (4, 6, 8–10). In general, CHM are considered naturally multifunctional and have minimal toxic side effects, low drug resistance, and reduced drug residue levels (11, 12). Chinese herbal feed additives (CHFA) are feed additives for animals that contain a combination of CHM. CHFA are becoming increasingly popular as a dietary supplement for animals due to their many beneficial effects (13, 14). Studies have found that they can improve the overall health and performance of animals (15, 16), as well as help to protect against some viruses’ infection and reduce their risk of diseases (17–22). Additionally, CHFA are cost-effective, making them an attractive option for farmers looking for animal feed additives. Furthermore, CHFA are an environmentally friendly alternative to traditional animal feed additives and can help reduce the amount of waste produced from animal feed production. Consequently, there has been increasing interest in the use of CHFA in animal nutrition (23, 24).

In recent years, the outbreak of African swine fever (ASF) and other highly contagious swine diseases has significantly impacted pig breeding and genetic resource preservation (25–28). To minimize disease transmission, artificial insemination is widely used in pig farming (29–31). However, the quality of boar sperm can be affected by factors such as storage time and management techniques. Traditional methods of improving sperm quality include breed selection and feeding management (32), but there is growing interest in the use of CHFA due to their significant benefits in animal nutrition. Studies have shown that CHM can improve the quality of boar sperm, including ejaculation volume, sperm motility, and reduce the sperm abnormality rate (33). Moreover, CHM is also used as an antioxidant for sperm preservation, with the addition of herbs such as resveratrol, icariin, tanshinone, rosmarinic acid, and Ganoderma lucidum polysaccharides prolonging sperm storage time and improving sperm quality (34). Thus, the use of CHFA in pig farming has garnered increasing attention for their potential to improve the quality and immune function of boar sperm, especially for imported boar semen.

China’s vast land and abundant resources provide unique opportunities for studying the effectiveness of CHMs as feed additives in improving the boar sperm quality (8). Due to the natural properties and multifunctional effects, CHMs hold great potential in improving the reproductive capacity of breeding boars and promoting the growth performance, antioxidant capacity, and intestinal microflora balance in weaned pigs (8, 35). Despite this potential, research on the use of CHFA for improving the quality of imported boar semen and reproductive capacity remains limited. To address this gap, we focus on the American Duroc boar as the research object and evaluating the impact of CHFA on breeding boar semen quality, serum reproductive hormone levels, as well as the reproductive capacity. To achieve this, relevant indicators of boar sperm quality will be assessed following the addition of CHFA to the daily diet of breeding boars. The findings of this study offer novel insights into the roles of CHMs in improving the production capacity of breeding boars.

## 2. Materials and methods

### 2.1. Experimental design and animals

In this experiment, 16 American Duroc boars were selected based on similar background including ages (~23 months), weights (~270 kg), and other aspects. The breeding boars were randomly allocated into two groups: the experimental group (8 boars) and the control group (8 boars), and the experiment was carried out accordingly. The animals were maintained in the laboratory animal center of Shanghai Academy of Agricultural Sciences and processed in compliance with the Shanghai Academy of Agricultural Sciences Guide for the Care and Use of Laboratory Animals. The animal experiments protocol was approved by the Animal Ethical Committee of Shanghai Academy of Agricultural Sciences, China (approval no. SAASPZ0522066).

### 2.2. Experimental and housing conditions

Each individual animal is housed in an adjacent pen of approximately 8 m^2^, which is equipped with an environmentally controlled slatted floor facility. During the entire experimental period (from August to November 2020), the animals had free access to feed and water, which were pro-vided via a self-feeder and nipple drinker, respectively. The temperature and humidity inside the pigsty were controlled within appropriate ranges during the experiment: the temperature was maintained between 16°C and 25°C, and the humidity was kept at 65%.

### 2.3. Chinese herbal feed additive and diets

The CHFA, which named “YiYangKang” (catalog no. 210509) was purchased from Hubei Jiulingcao Biotechnology Inc., Hubei, China. It is a powder extract mainly comprises Epimedium, Astragalus, Codonopsis, Atractylodes, and other ingredients. A basal diet without antibiotics was purchased from CARGILL FEED JIAXING Ltd. Co., Jiaxing, China. Throughout the entire experimental period, the control group received the normal diet, while the experimental group (CHFA treated group) received the normal diet supplemented with 1 kg/ton of CHFA according to the manufacturer’s instruction.

### 2.4. Feeding procedures

The feeding method for the experimental and control groups of breeding boars followed the standard feeding protocols for normal boars. Specifically, boars were fed twice daily at regular times, provided with free access to water, and exercised for approximately 2 kilometers.

### 2.5. Semen collection and assessing sperm viability and abnormality

During the experiment, all boars underwent normal semen collection procedures, as described in the previous studies (36–38). Briefly, semen samples were collected weekly for a total of 16 times from the 16 pigs in the control and CHFA treated groups. Semen was collected routinely by artificial vagina technique (pre-warmed at 42°C) by the same qualified researchers. After semen collection, semen was transferred directly into the laboratory. Then, the volume (mL), sperm concentration (billion/mL), sperm motility (%), and sperm abnormality rate (%) were recorded during each collection. The sperm parameters were analyzed using a specialized sperm detection analyzer (model XY-18) developed by Shanghai Xiangxin Livestock Ltd. Co., Shanghai, China.

### 2.6. Enzyme-linked immunosorbent assay

To investigate the variations in reproductive hormone levels in boars, we collected venous blood samples before and after feeding them with CHFA. Throughout the experimental period, we continued to collect blood samples every 7 days. Subsequently, the serum was separated after centrifugation (2,000 rpm, 30 min, 4°C) and stored at-80°C, and the serum concentrations of reproductive hormones, including follicle-stimulating hormone (FSH), luteinizing hormone (LH), and testosterone (T) were quantified using ELISA kits (Shanghai Keshun Biotechnology Inc., Shanghai, China), as described in the method by Li et al. (36).

### 2.7. Boar reproductive capacity assessment

To evaluate the impact of the CHFA on boar reproductive capacity, we conducted a study using Large White sows (~13 months old) in a natural estrus state that lasted approximately 21 days. During the estrus cycle, we used the collected sperm samples for artificial insemination of binary sows. Subsequently, the litter size at birth, number of live births, number of 28-day weaned piglets, as well as the birth weight and 28-day weaning weight of piglets were measured and evaluated throughout the experiment.

### 2.8. Statistical analysis

Statistical analysis was performed using GraphPad Prism 8.0 software (GraphPad Software Inc., San Diego CA). The statistical significance was calculated using student’s t-test, and the group treated with the herbal feed additive was compared to the control group. Each measurement was performed at least three independent experiments, and the results were presented as mean ± standard deviation (SD). Statistical significance was defined as a *p* < 0.05 and high statistical significance was defined as *p* < 0.01, 0.001 or 0.0001.

## 3. Results

### 3.1. The influence of CHFA on the nutrient content of the breeding boar diet

To eliminate the differences in nutrient content of the boar diet between the control and experimental groups, as well as the effect of CHFA on the nutrient content of the boar diet, we first randomly sampled the boar diets before and after the addition of the CHFA and determined the relevant nutrient content. The nutrient content of the two types of diet is shown in Table 1, which indicates that the addition of the CHFA has no significant effect on the nutrient content of the boar diets, which suggests that the use of CHFA as a feed additive for breeding boars does not affect the overall nutrient composition of their diet.

**Table 1:** Ingredient composition and analyzed nutrient contents of breeding boars’ diet.

Nutrition	Animal groups	
	Control	CHFA
Crude protein (%)	17.90	18.00
Digestive energy (MJ/kg)	13.70	14.10
Calcium (%)	0.65	0.65
Total phosphorus (%)	0.49	0.49
Lysine (%)	0.85	0.86
Methionine (%)	0.29	0.28
Threonine (%)	0.56	0.61
Glutamic acid (%)	2.91	3.12
Coarse fiber (%)	3.00	3.00

CHFA, Chinese herb feed additive.

### 3.2. CHFA can promote the boar sperm production

**Figure 1:**
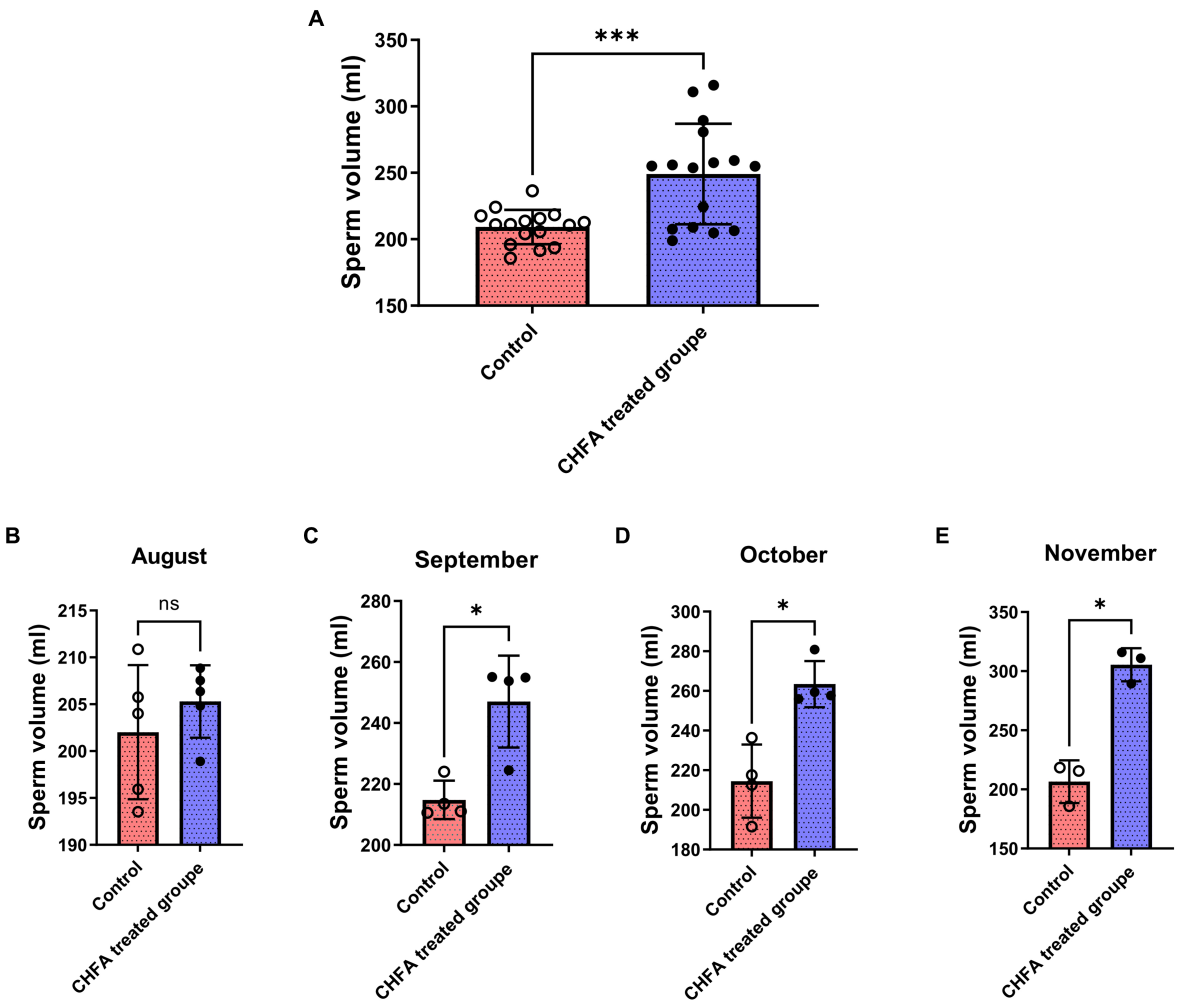
Effects of CHFA as a dietary additive on sperm production in breeding boars. **(A)** Depicts the overall effect of CHFA on sperm production in breeding boars. **(B–E)** Show the specific effects of CHFA on sperm production in breeding boars during different months (August, September, October, and November, respectively) compared to the control group. The x-axis represents different treatment groups, while the y-axis represents sperm production volume (*n* = 8/group). “*” means *p* < 0.05; “***” means *p* < 0.001. ns means not significant.

To investigate the potential impact of CHFA on boar sperm production, we tracked and analyzed the boar sperm volume over a 4-month experimental period from August to November (Figure 1). We found that the total sperm volume of control and CHFA treated groups were 209.2 ± 12.94 (mean ± SD), and 249.0 ± 37.85, respectively (Table 2). This result proved that the breeding boars of CHFA treated group had a significantly higher sperm volume than the ones in control group (Figure 1A). Further analysis showed that although there was no significant difference in sperm volume between the control and experimental groups in August (Figure 1B), the CHFA treated group consistently had a significantly higher sperm volume in the other 3 months (Figures 1C,D). These findings suggest that the sperm volume of boars may be positively influenced by the addition of CHFA to their diet, with the CHFA treated group showing a continuous increase in sperm volume compared to the control group throughout the experimental period (Table 2). Notably, the nutrient content of the boar feed was not affected by the addition of CHFA, indicating that this increase in sperm volume was not due to changes in the overall nutrient composition of the diet.

**Table 2:** Effects of Chinese herb feed additive treatments on the sperm quality parameters of breeding boars.

Items		Volume (ml)	Concentration (10^9^)	Motility (%)	Abnormality (%)
August	Control	202.0 ± 7.159	3.460 ± 0.2711	62.57 ± 1.720	17.47 ± 0.2654
	CHFA	205.3 ± 3.863	3.454 ± 0.1518	69.60 ± 3.360**	16.21 ± 0.1082***
September	Control	214.8 ± 6.299	3.958 ± 0.5266	67.70 ± 2.318	17.47 ± 0.3823
	CHFA	247.0 ± 15.06*	4.503 ± 0.2901*	77.37 ± 1.669**	15.89 ± 0.0379**
October	Control	214.5 ± 18.42	4.043 ± 0.0359	76.90 ± 1.409	17.24 ± 0.6910
	CHFA	263.4 ± 11.67*	4.533 ± 0.1282**	80.26 ± 1.460*	14.53 ± 0.3380**
November	Control	206.6 ± 18.10	4.357 ± 0.2301	77.38 ± 1.617	17.81 ± 0.1172
	CHFA	305.4 ± 14.06*	5.147 ± 0.0750*	84.57 ± 1.121*	11.35 ± 0.9808**
Total	Control	209.2 ± 12.94	3.898 ± 0.4415	70.21 ± 6.782	17.48 ± 0.4274
	CHFA	249.0 ± 37.85***	4.303 ± 0.6590**	77.01 ± 6.061***	14.80 ± 1.878***

CHFA, Chinese herb feed additive; “*” means *p* < 0.05; “**” means *p* < 0.01; “***” means *p* < 0.001.

### 3.3. CHFA can significantly increase the boar sperm concentration

**Figure 2:**
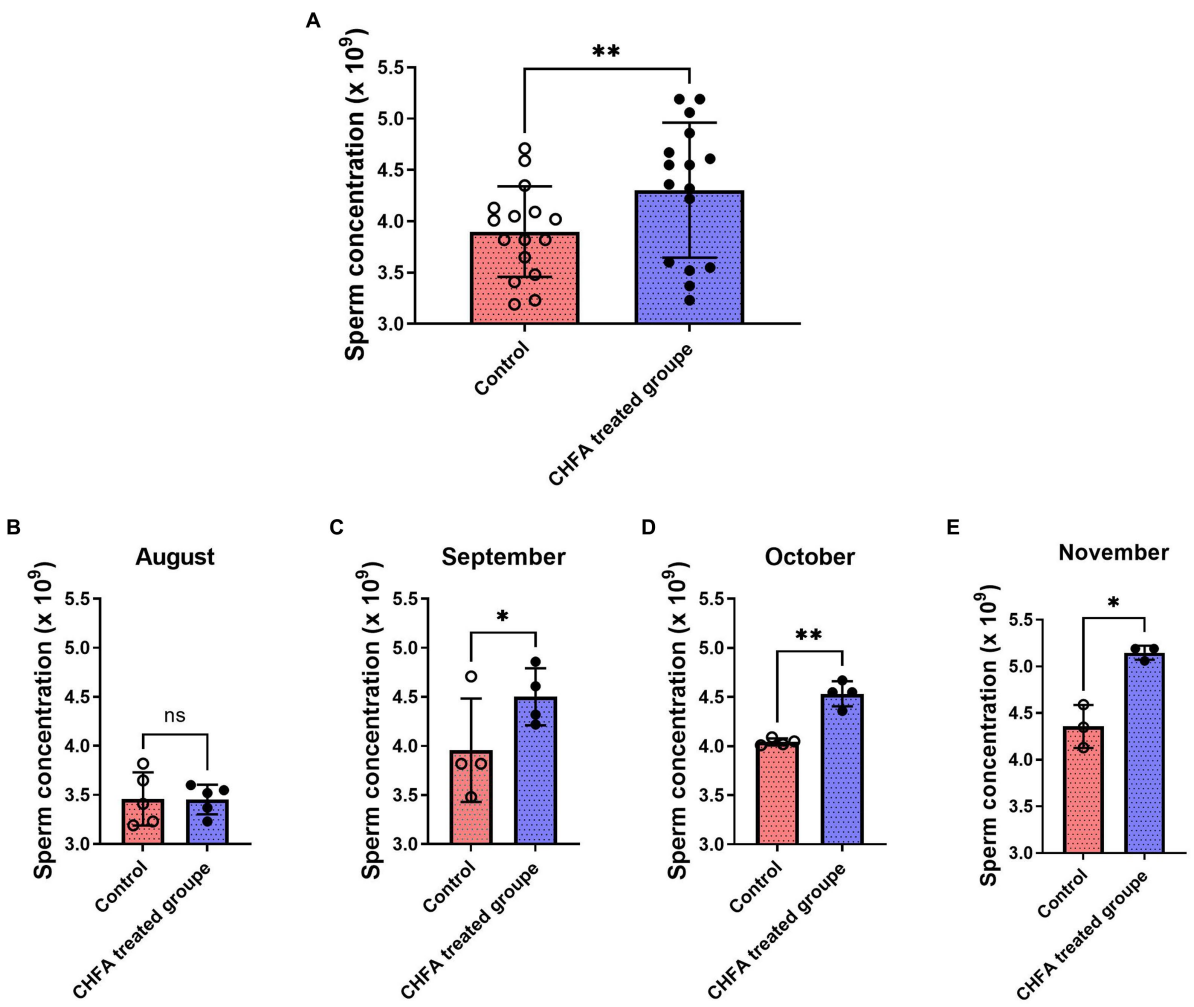
Effects of CHFA as a dietary additive on sperm concentration in breeding boars. **(A)** Depicts the overall effect of CHFA on sperm concentration in breeding boars. **(B–E)** Show the specific effects of CHFA on sperm concentration in breeding boars during different months (August, September, October, and November, respectively) compared to the control group. The x-axis represents different treatment groups, while the y-axis represents the sperm concentration (*n* = 8/group). “*” means *p* < 0.05; “**” means *p* < 0.01. ns means not significant.

Sperm concentration is an important indicator of the sperm quality. In order to investigate the effect of CHFA on boar sperm concentration, we conducted concentration analysis on the collected sperm samples. Similar to the method of measuring the boar sperm volume, we tracked and analyzed the sperm concentration of the control group and the experimental group of male breeding pigs during the four-month experimental period. We found that the total sperm concentration of control and CHFA treated groups were 3.898 ± 0.4415 (mean ± SD), and 4.303 ± 0.6590, respectively (Table 2). This result showed that compared with the control group, the boar sperm concentration in the CHFA group was significantly higher (*p* = 0.0011; Figure 2A). To clarify whether boar sperm concentration changed with the increasing addition of CHFA, we further analyzed the changes in boar sperm concentration during the experimental period. The results showed that except August, where has no significant difference in sperm concentration between the CHFA treated group and the control group of the boars (*p* = 0.9729; Figure 2B). However, in the remaining three months, the boar sperm concentration in the CHFA treated group was significantly higher than that in the control group (September, *p* = 0.0336; October, *p* = 0.0040; November, *p* = 0.0443; Figures 2C,D). These results suggest that boar sperm concentration is positively associated with CHFA addition (Table 2). In other words, compared to the control group of boars without CHFA addition, the boar sperm concentration in the experimental group continued to increase with the increasing addition of CHFA over time.

### 3.4. CHFA can significantly enhance the boar sperm motility

**Figure 3:**
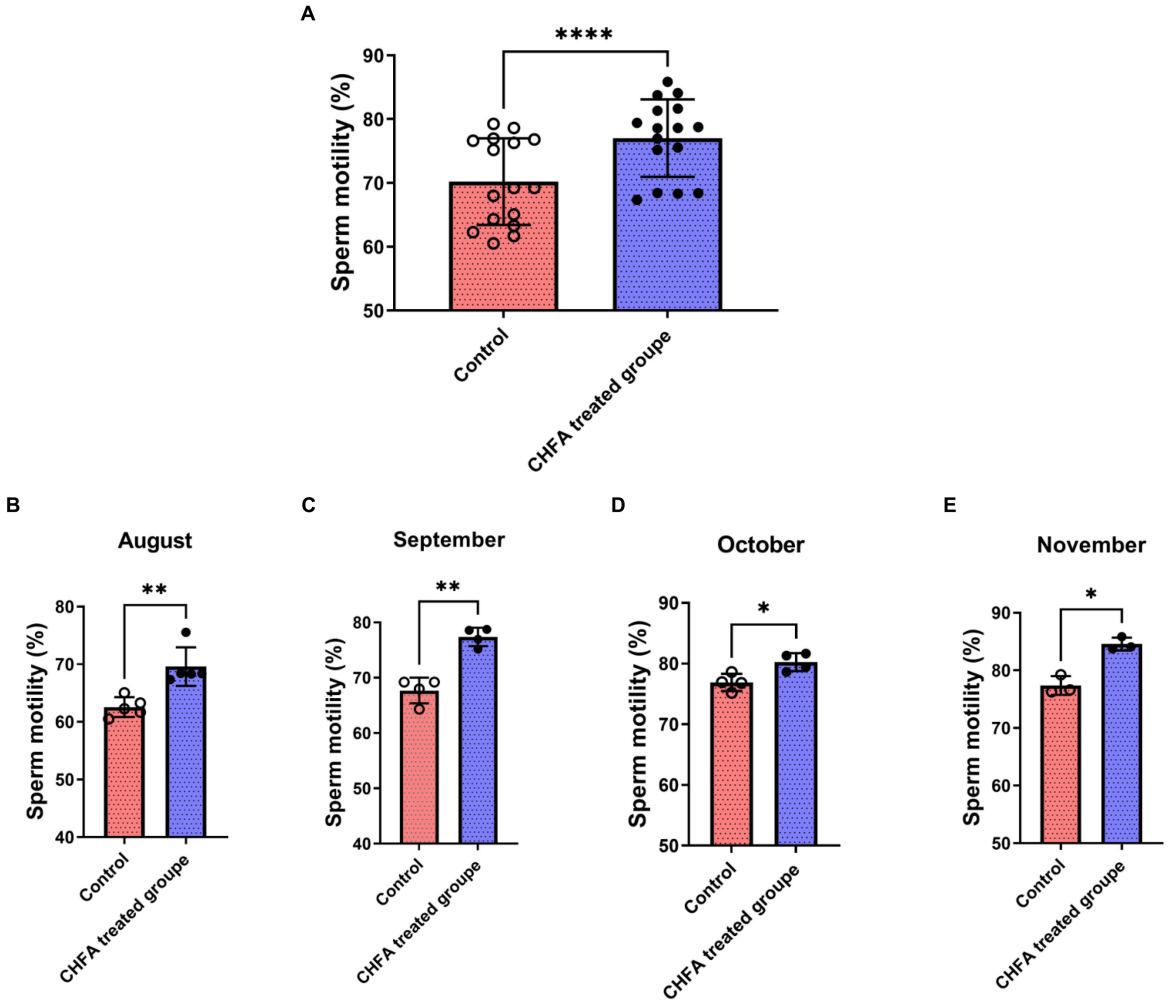
Effects of CHFA as a dietary additive on sperm motility in breeding boars. **(A)** Depicts the overall effect of CHFA on sperm motility in breeding boars. **(B–E)** Show the specific effects of CHFA on sperm motility in breeding boars during different months (August, September, October, and November, respectively) compared to the control group. The x-axis represents different treatment groups, while the y-axis represents the sperm motility (*n* = 8/group). “*” means *p* < 0.05; “**” means *p* < 0.01; “****” means *p* < 0.0001.

Sperm motility, which refers to the percentage of forward-moving sperm in semen, is a critical indicator of sperm quality. In boars, sperm motility is closely associated with the fertility rate of female pigs and is, therefore, a primary factor for evaluating semen quality. In order to investigate the effect of CHFA on sperm motility in boars, we further measured the sperm motility of the control group and the CHFA treated group of boars. We tracked and analyzed the boar sperm motility in both groups during the four-month experimental period from August to November. We found that the total sperm motility of control and CHFA treated groups were 70.21 ± 6.782 (mean ± SD), and 77.01 ± 6.061, respectively (Table 2). This result showed that the boar sperm motility in the CHFA treated group was significantly higher compared to the control group (*p* < 0.0001; Figure 3A). Further analysis showed that the boar sperm motility in the CHFA treated group was significantly higher than that in the control group during the experimental period (August, *p* = 0.0016; September, *p* = 0.0099; October, *p* = 0.0183; November, *p* = 0.0391; Figures 3C,D). These results indicate that CHFA can significantly enhance the boar sperm motility.

### 3.5. CHFA addition can significantly reduce the sperm abnormality rate

**Figure 4:**
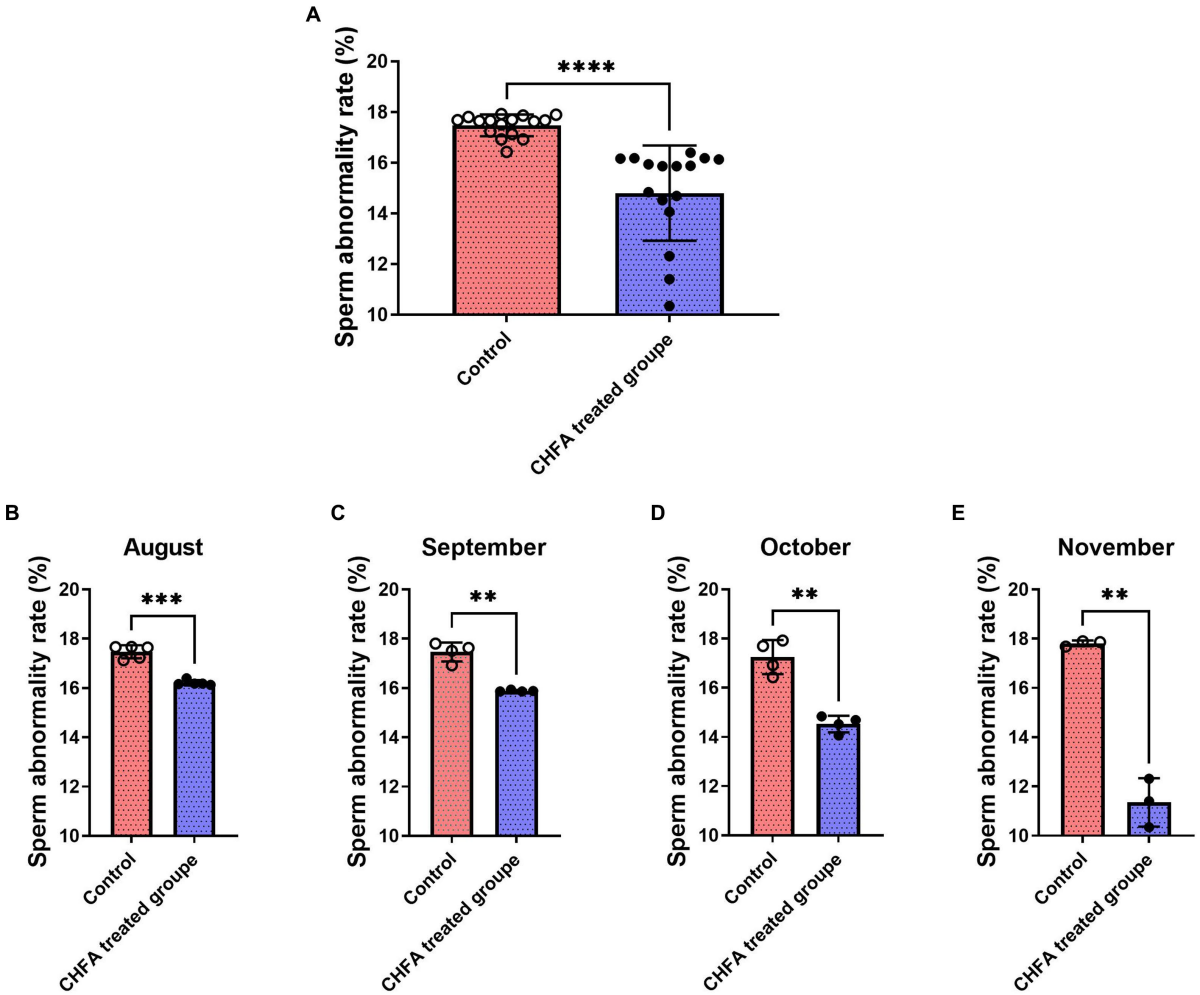
Effects of CHFA as a dietary additive on sperm abnormalities in breeding boars. **(A)** Depicts the overall effect of CHFA on sperm abnormalities in breeding boars. **(B–E)** Show the specific effects of CHFA on sperm abnormalities in breeding boars during different months (August, September, October, and November, respectively) compared to the control group. The x-axis represents different treatment groups, while the y-axis represents the sperm abnormalities (*n* = 8/group). “**” means *p* < 0.01; “***” means *p* < 0.001; “****” means *p* < 0.0001.

Sperm abnormality rate is the proportion of abnormal sperm to total sperm, where abnormal sperm refers to those with abnormal morphology or function. Abnormal sperm may not fertilize normally or produce healthy offspring. The abnormal sperm rate of boars is an important indicator for evaluating their reproductive capacity and fertility. Generally, the lower the sperm abnormality rate of boars, the stronger their reproductive ability, and the healthier their offspring. In order to investigate the effect of CHFA on the sperm abnormality rate in breeding boars, we measured the abnormality rate of semen samples collected from the control group and the CHFA treated group. The sperm abnormality rate in both groups of breeding boars was tracked and analyzed during the 4-month experimental period (August, September, October, and November). We found that the total sperm abnormality rate of control and CHFA treated groups were 17.48 ± 0.4274 (mean ± SD), and 14.80 ± 1.878, respectively (Table 2). This result showed that the sperm abnormality rate in breeding boars with CHFA addition was significantly lower than that in the control group (*p* < 0.0001; Figure 4A). Further results showed that during each month, the sperm abnormality rate in breeding boars with CHFA addition was significantly lower than that in the control group (August, *p* = 0.0004; September, *p* = 0.0039; October, *p* = 0.0089; November, *p* = 0.0060; Figures 4C,D). These results indicate that CHFA can significantly reduce the sperm abnormality rate in breeding boars.

### 3.6. CHFA addition promotes the serum reproductive hormone levels

**Figure 5:**
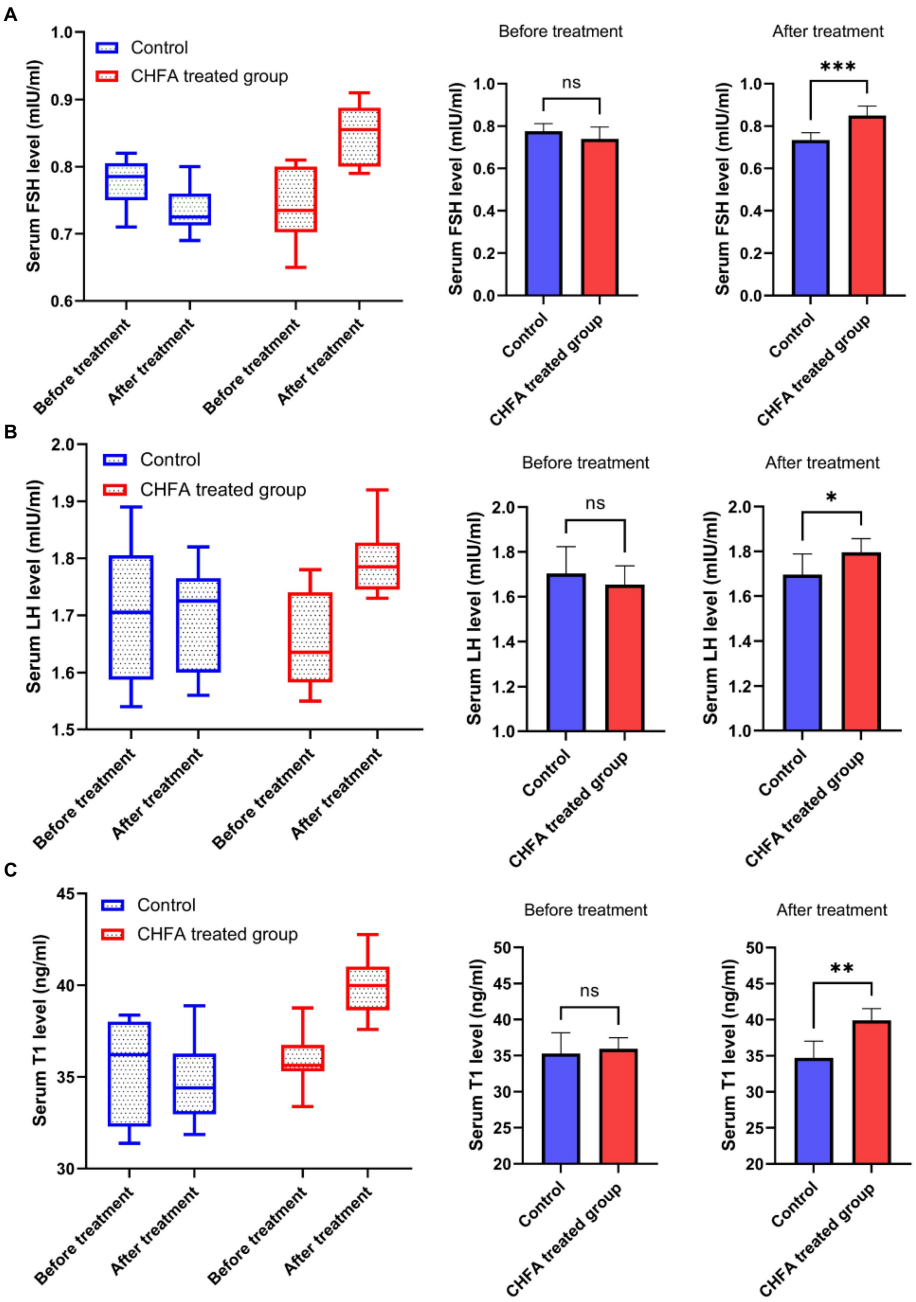
CHFA significantly increased the serum levels of FSH, LH, and T in breeding boars. The study measured the serum levels of these hormones, including **(A)**, LH **(B)**, and T **(C)** in breeding boars before and after CHFA addition, with the x-axis representing the different groups and the y-axis representing the level of serum reproductive hormones. “*” means *p* < 0.05; “**” means *p* < 0.01; “***” means *p* < 0.001.

The serum reproductive hormone levels in breeding boars provide valuable insights into their growth performance, nutrition, and development. By measuring these hormone levels, pig farms can optimize feeding management measures. In order to clarify the effect of the CHFA on the serum reproductive hormone levels of breeding boars, we next detect the reproductive hormone levels (including follicle-stimulating hormone, FSH; luteinizing hormone, LH; and testosterone, T) in the serum of breeding boars (Figure 5). We found that the FSH level in the control group was 0.7750 ± 0.0363 (mean ± SD) before CHFA treatment and 0.7338 ± 0.0350 after CHFA treatment, the LH level was 1.704 ± 0.1203 before CHFA treatment and 1.696 ± 0.0923 after CHFA treatment, and the testosterone level was 35.29 ± 2.867 before CHFA treatment and 34.71 ± 2.289 after CHFA treatment, respectively (Table 3). These results suggested that there was no significant change in the levels of FSH (*p* = 0.1443) (Figure 5A), LH (*p* = 0.4595; Figure 5B), and testosterone (*p* = 0.5066; Figure 5C) in the serum of breeding boars in the control group throughout the entire experimental period. However, after CHFA was added to the diet of breeding boars, the content of FSH (*p* < 0.0005; Figure 5A), LH (*p* < 0.0181; Figure 5B), and testosterone (*p* < 0.0027; Figure 5C) in their serum significantly increased compared to before the CHFA addition (Table 3). This result indicates that adding CHFA to the diet can significantly promote the serum contents of FSH, LH, and testosterone in breeding boars.

**Table 3:** Effects of Chinese herb feed additive treatments on the serum reproductive hormone parameters of breeding boars.

Items		Before or after CHFA treatment	
		Before CHFA treatment	After CHFA treatment
FSH (mIU/ml)	Control	0.7750 ± 0.0363	0.7338 ± 0.0350
	CHFA	0.7400 ± 0.0558	0.8500 ± 0.0444***
LH (ng/ml)	Control	1.704 ± 0.1203	1.696 ± 0.0923
	CHFA	1.654 ± 0.0843	1.796 ± 0.0612*
Testosterone (mIU/ml)	Control	35.29 ± 2.867	34.71 ± 2.289
	CHFA	35.95 ± 1.536	39.91 ± 1.620**

CHFA, Chinese herb feed additive; FSH, follicle-stimulating hormone; LH, luteinizing hormone; T, testosterone. “*” means *p* < 0.05; “**” means *p* < 0.01; “***” means *p* < 0.001.

### 3.7. CHFA addition enhance breeding boar’s reproductive capacity when mating with sows

**Figure 6:**
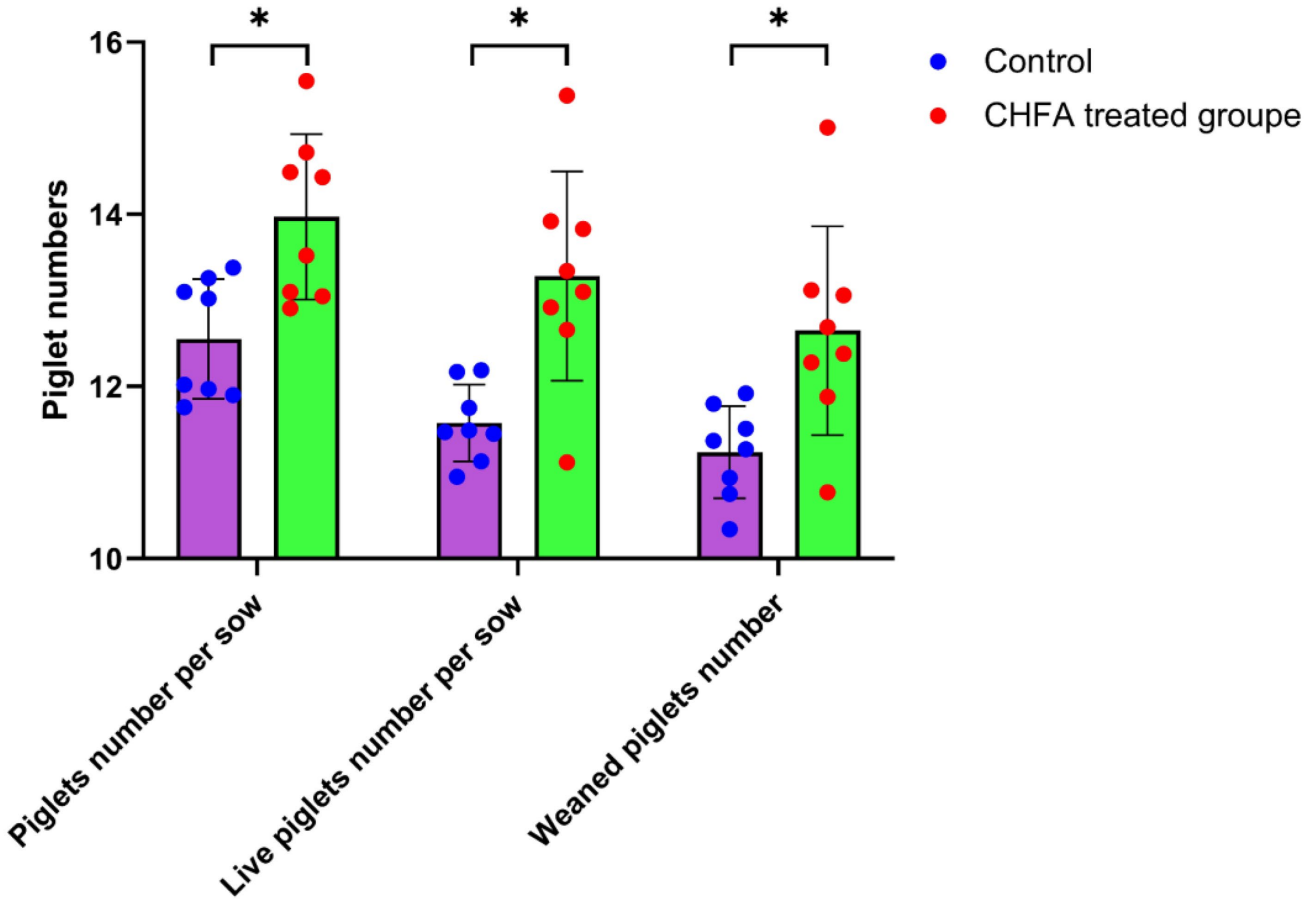
CHFA addition increase the reproductive capacity of breeding boars when mating with sows. In this study, the experimental group of boars was provided with feed supplemented with CHFA, while the control group was fed a basic diet without CHFA. Semen samples were collected from both groups of boars for insemination of sows, and reproductive performance was evaluated by measuring piglet number per sow, live piglet number per sow, and weaned piglet number at 28 days old. The x-axis represents piglet per sow, live piglet per sow, and weaned piglets for the control and CHFA treated groups of boars after insemination with sows, while the y-axis represents the number of piglets delivered. The results show that CHFA significantly improves the reproductive performance of boars when mating with sows. “*” means *p* < 0.05.

The litter size, number of live births, and number of piglets weaned at 28 days old are important indicators of sow reproductive capacity, which is closely related to the quality of boar sperm. Our study has demonstrated that the addition of CHFA to the diet of boars can significantly improve sperm quality and hormone levels in breeding boars, which suggests that using sperm from these breeding boars can result in more litters and better piglet production performance. To confirm the impact of adding CHFA to the diet of boars on the reproductive capacity of breeding sows, we mated 120 dual-element sows with boars in both the control and CHFA treated groups. We then recorded the litter size, number of live births, and number of piglets weaned at 28 days old for each dual-element sow after mating. The results revealed that adding CHFA to the diet of boars significantly increased the litter size, number of live births, and number of piglets weaned at 28 days old of the sows (Figure 6). Therefore, it can be concluded that the addition of CHFA to the diet of boars can significantly enhance the reproductive capacity of breeding sows when using sperm from these breeding boars.

### 3.8. The effects of the CHFA on the average birth weight and weight gain of piglets

**Figure 7:**
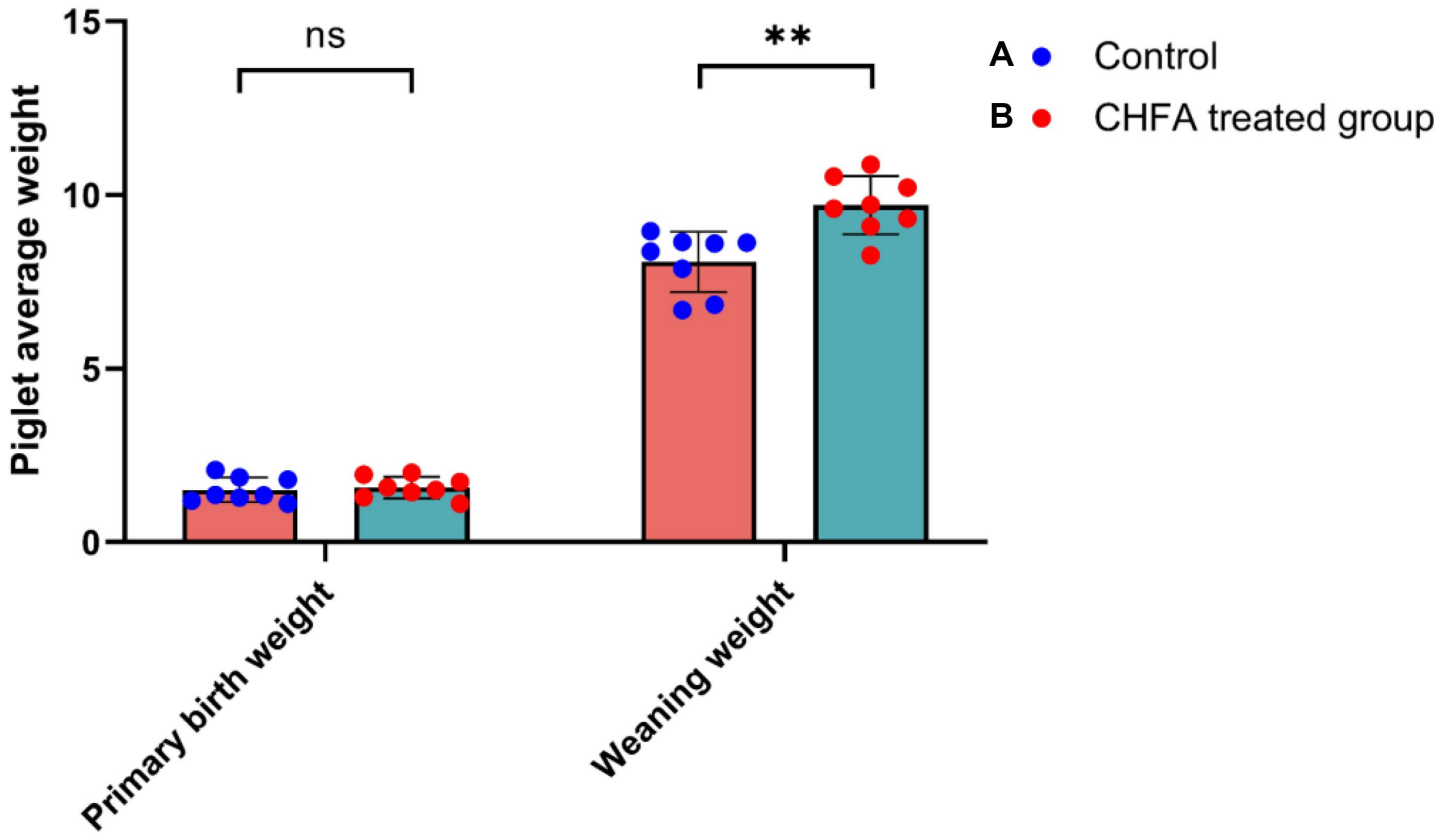
CHFA addition can significantly enhance the piglets’ weaning weight born to sows inseminated with sperm from these breeding boars. The control group **(A)** was fed with a basic diet without CHFA, while the experimental group **(B)** of breeding boars was fed with CHFA-supplemented feed. Sperm was collected from both groups of boars to inseminate sows, and the growth performance of their offspring was evaluated by measuring the average weight of piglets at day 28 (piglet average weight). The results were used to assess the effect of CHFA supplementation on the growth performance of the boar offspring. The x-axis represents the two weight parameters (primary birth weight or weaning weight), and the y-axis represents the average weight of the piglet. “**” means *p* < 0.01. ns means not significant.

**Figure 8:**
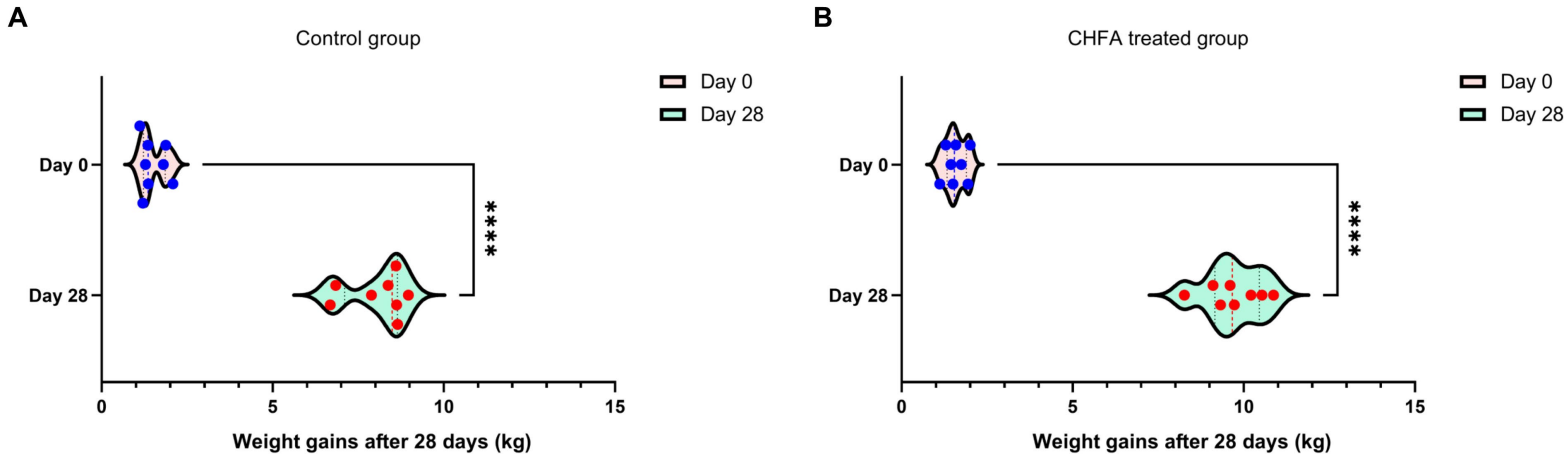
CHFA addition can significantly enhance the weight gain of piglets born to sows inseminated with sperm from these breeding boars. The control group **(A)** was fed with a basic diet without CHFA, while the experimental group **(B)** of breeding boars was fed with CHFA-supplemented feed. Sperm was collected from both groups of boars to inseminate sows, and the weight gain (weaning weight minus primary birth weight) of their offspring in each group was calculated. The results were used to assess the effect of CHFA supplementation on the weight gain of the boar offspring. The x-axis represents the weight gain at 28 days after birth, and the y-axis represents the time of birth (day 0) or weaning at 28 days (day 28). “****” means *p* < 0.0001.

In order to clarify the impact of adding CHFA to the diet of breeding boars on the average birth weight and weight gain of piglets born to sows inseminated with serum from these boars, we analyzed the differences in these parameters between sows in the control and CHFA treated groups. The results showed that there was no significant difference in the average birth weight of piglets born to sows inseminated with serum from either group (Figure 7). However, the weaning weight of piglets born to sows inseminated with serum from the CHFA treated group was significantly higher than that of piglets born to sows inseminated with serum from the control group (Figure 7). We further analyzed the difference in weight gain between the weaning weight and birth weight of piglets born to sows in both the control and CHFA treated groups at 28 days after birth. The results showed that although the weight gain of piglets born to sows inseminated with serum from both groups increased significantly, the increase amount in weight gain for the CHFA treated group was greater than that of the control group (Figure 8). These findings suggest that adding CHFA to the diet of boars can promote the weight gain of piglets born to sows inseminated with sperm from these breeding boars.

## 4. Discussion

Recently, the artificial insemination (AI) has been extensively used in swine industry to leverage the superior genetic background and enhanced fertility of boars and sows (39–42). In the current context of normalizing African swine fever (ASF) in China, AI is widely adopted for both large- and small-scale pig farms to minimize disease transmission. The introduction of new breeds often involves the use of imported boar sperm, leading to an increase in the frequency of boar sperm collection, which directly affects the health status of piglets. Previous studies have shown that some extracts from CHM or chemical substances have protective effects on fresh sperm (43, 44). Hashem et al. found that feeding male rabbits with plant extracts significantly enhanced the sperm quality and antioxidant capacity, mainly through high-density lipoprotein transporting cholesterol to the steroid-producing tissue similar to the testis, and eliminating excess low-density lipoprotein through the liver (45). Zhou and co-workers demonstrated that adding taxifolin (TAX) to the basal diet could improve boar semen quality by increasing sperm motility and sperm concentration (46). Another study reported that dietary supplementation of antioxidants, such as lysine and L-arginine, could improve sperm quality in boars (41, 42). In addition, a study found that herbal supplements, such as nettle and rosehip, could improve the sperm quality of boars (47). However, further research is needed to determine the long-term safety and optimal use of these additives in improving the quality of boar sperm, and whether this protective effect has a beneficial impact on sow reproductive capacity.

Although the above-mentioned researches indicate that the trace element and CHFA can improve sperm quality in boars, there is a lack of systematic research on the related effects on the sperm vitality, concentration, and sperm abnormality rate of the imported breeding boars. In order to elucidate the effect of CHFA on the sperm quality of imported breeding boars and to mitigate the deleterious effects of low temperature on boar activity and sperm production, we focused on Duroc boars and conducted experiments during the transition from summer to autumn. The Duroc boars were administered CHFA for an extended period of 4 months, surpassing the growth cycle of boar sperm from spermatogonial cells to mature sperm which is approximately 49 days. The findings obviously demonstrated that CHFA administration significantly enhanced the semen volume, motility, and sperm concentration, concurrently with a marked reduction in the sperm abnormality rate among boars. These findings suggest that CHFA could be used as a promising additive for improving the reproductive performance of boars, especially for imported boars in the context of ASF normalization in China.

The pituitary gland secretes two important hormones, FSH and LH, which play a crucial role in the hypothalamic–pituitary-gonadal (HPG) axis (48–51). FSH, a glycoprotein hormone secreted by the anterior pituitary acidophilic cells, regulates a series of physiological processes including development, growth, puberty, and reproduction-related functions (52–54). LH, on the other hand, synergistically contributes to reproductive-related physiological processes (55, 56). FSH and LH act on the seminiferous tubules of the testes to promote sperm formation (57). CHMs are effective in improving the quality of boar sperm, mainly because these herbs contain components such as flavonoids, Epimedium, and Cistanche which not only regulate the body’s hormone levels but also inhibit apoptosis of germ cells, thereby regulating reproductive function, enhancing the body’s immune system, and modulating antioxidative activity (33, 58, 59). Testosterone provides a superior growth environment for sperm formation by regulating interstitial and supporting cells, stimulating sperm production, and improving sperm quality parameters such as vitality and concentration (60, 61). In a study by Yun et al., feeding pigs with ginger extract resulted in a significant increase in serum testosterone levels in boars, possibly due to the extract’s direct impact on the testicular interstitial cells (62). There are other reports state that Chinese herbs can regulate the hormone levels of animals and improve their reproductive performance (63, 64). In addition, CHFA addition can also improve the growth performance, meat quality, and nutrient digestibility parameters of pigs (8). In this study, a CHFA composed of Epimedium, Astragalus, Codonopsis, Atractylodes, and other herbs significantly increased the serum follicle-stimulating hormone (Figure 5A) and luteinizing hormone (Figure 5B), and extensively increased the testosterone levels of experimental boars (Figure 5C), thereby explaining the reason for the increase in semen volume (Figure 1), concentration (Figure 2), and motility (Figure 3), and the decrease in sperm abnormality rate in boars fed with the CHFA (Figure 4). This phenomenon is consistent with the reproductive mechanism of the HPG axis in regulating sperm production (65). These findings suggest that CHFA addition may have a positive impact on the reproductive performance of breeding boars. Further research is needed to determine the mechanism behind this effect and to optimize the use of CHFA in pig breeding.

The breeding boars’ reproductive capacity is primarily related to the quality of their sperm, while indicators such as litter size, number of live-born piglets, weaning piglet count, birth weight, weaning weight, and lactation ability reflect the reproductive capacity of sows. Studies had shown that phytogenic additives are being explored for maternal supplementation in sows with the aim of augmenting the litter size. Additionally, such supplementation may have implications for the gastrointestinal tract health of both the sow and the nursed piglets (66). Another study showed that dietary supplementation with modified Bazhen powder (MBP) improved the reproductive performance, serum traits, and immune status of sows, as well as changing breast milk microbes and metabolome characterization (67). All of the aforementioned studies have examined the direct effects of CHFA on the reproductive capacity of sows, but no research has yet been conducted on the effects of such additives on the reproductive capacity of boars used for artificial insemination of sows. Our study represents the first investigation into the impact of feeding a CHFA to boars on the reproductive capacity of inseminated sows, thus indirectly reflecting the effects of such additives on the production performance of breeding boars. We found that administering the CHFA significantly improved the quality of boar sperm, resulting in a notable increase in the average litter size, number of live-born piglets, weaning piglet count at 28 days, and weaning weight at 28 days, compared to the control group. Therefore, the CHFA not only directly improves the quality of boar sperm, but also significantly promotes the average birth weight and weight gain of piglets born to sows inseminated with sperm from these breeding boars.

## 5. Conclusion

This study indicates that CHFA can significantly increase the sperm production volume and improve the sperm viability, concentration, and extensively reduce the sperm abnormality rate of the breeding boars, reflecting the good protective effect on the sperm of the breeding boars. In addition, CHFA can significantly increase the reproductive hormones content, especially follicle-stimulating hormone (FSH), luteinizing hormone (LH), and testosterone (T) in the serum of the breeding boars. This provides new evidence for the reproductive mechanism of regulating sperm production in the HPG axis. Furthermore, the CHFA can enhance the boar sperm quality, ultimately improving the average number of piglets born, the average number of piglets born alive, the number of piglets weaned at 28 days, and the weaning weight at 28 days by improving the sperm quality of the breeding boars. In summary, CHFA can improve the sperm quality of imported breeding boars and their reproductive capacity with breeding sows, suggesting that CHFA have potential application prospects in improving the reproductive capacity of imported animals.

## Data availability statement

The original contributions presented in the study are included in the article/supplementary material, further inquiries can be directed to the corresponding authors.

## Ethics statement

The animal study was reviewed and approved by Animal Ethical Committee of Shanghai Academy of Agricultural Sciences, China (approval ID: SAASPZ0522066).

## Author contributions

WT and YT: conceptualization. WT and JH: methodology. YZ and XL: software. WT and WZ: validation and writing – review and editing. WT and XL: formal analysis. WT, WZ, HW, and YZ: investigation. YT: resources and funding acquisition. WT and BL: data curation. WT: writing – original draft and visualization. YT and XW: supervision. All authors contributed to the article and approved the submitted version.

## Funding

This work was funded by the Shanghai Key Project on Agricultural Development (grant no. 2021-02-08-00-12-F00768) and the Shanghai Academy of Agricultural Sciences (SAAS) Program for Excellent Research Team (grant no. 2022-B-016).

## Conflict of interest

The authors declare that the research was conducted in the absence of any commercial or financial relationships that could be construed as a potential conflict of interest.

## Publisher’s note

All claims expressed in this article are solely those of the authors and do not necessarily represent those of their affiliated organizations, or those of the publisher, the editors and the reviewers. Any product that may be evaluated in this article, or claim that may be made by its manufacturer, is not guaranteed or endorsed by the publisher.
